# Transmembrane protein 120A (TMEM-120A/TACAN) coordinates with PIEZO channel during *Caenorhabditis elegans* reproductive regulation

**DOI:** 10.1093/g3journal/jkad251

**Published:** 2023-12-05

**Authors:** Xiaofei Bai, Andy Golden

**Affiliations:** Department of Biology, University of Florida, Gainesville, FL 32610, USA; Genetics Institute, University of Florida, Gainesville, FL 32610, USA; National Institute of Diabetes and Digestive and Kidney Diseases, National Institutes of Health, Bethesda, MD 20892, USA; National Institute of Diabetes and Digestive and Kidney Diseases, National Institutes of Health, Bethesda, MD 20892, USA

**Keywords:** TACAN, PIEZO, *C. elegans*, CRISPR/Cas9, fertility, reproduction, genetics

## Abstract

Membrane protein TMEM120A (also known as TACAN) was presumed to be both a mechanically activated molecule and a lipid-modifying enzyme. TMEM120A has been identified as a negative regulator of the essential excitatory mechanosensitive protein PIEZO2. However, the extent to which TMEM120A mediates PIEZO2's activity during physiological processes remains largely unknown. In this study, we used the *Caenorhabditis elegans* reproductive tract to explore the functional contribution of *tmem-120*, the sole *TMEM120A/B* ortholog, and its genetic interaction with *pezo-1* in vivo. *tmem-120* was expressed throughout the *C. elegans* development, particularly in the germline, embryos, and spermatheca. A *tmem-120* mutant with a full-length deletion (*tmem-120Δ*) displayed deformed germline, maternal sterility, and a reduced brood size. In vivo live imaging revealed that pinched zygotes were frequently observed in the uterus of *tmem-120Δ* mutant animals, suggesting damage during spermathecal contraction. We then employed the auxin-inducible degradation system to degrade TMEM-120 protein in all somatic tissues or the germline, both of which resulted in reduced brood sizes. These findings suggested that multiple inputs of *tmem-120* from different tissues regulate reproduction. Lastly, the loss of *tmem-120* alleviated the brood size reduction and defective sperm navigation behavior in the *pezo-1Δ* mutant. Overall, our findings reveal a role for *tmem-120* in regulating reproductive physiology in *C. elegans*, and suggest an epistatic interaction between *pezo-1* and *tmem-120* when governing proper reproduction.

## Introduction

PIEZOs, including PIEZO1 and PIEZO2, are excitatory mechanosensitive proteins. In humans, both PIEZO1 and PIEZO2 play essential roles in mechanosensation throughout development and in various physiological processes, such as touch, hearing, proprioception, and reproductive performance (Coste et al. 2010; Woo et al. 2015; Wang et al. 2016; Li et al. 2021; Lam et al. 2023). Loss-of-function or gain-of-function mutations in *PIEZOs* have been linked to a spectrum of physiological disorders and diseases. These conditions include muscular dystrophy, proprioception issues, lymphatic dysplasia, hereditary stomatocytosis, respiratory distress, arthrogryposis, skeletal and bone abnormalities, and urinary system-related voiding symptoms (Coste et al. 2010, Albuisson et al. 2013; Andolfo et al. 2013; Bae et al. 2013; Coste et al. 2013; Fotiou et al. 2015; Lukacs et al. 2015; Chesler et al. 2016; Alper 2017; Murthy et al. 2017; Nonomura et al. 2017; Del Marmol et al. 2018; Marshall et al. 2020). Despite significant advances in understanding the electrophysiological characteristics of PIEZOs at the cellular membrane and gaining insights into the structural aspects of PIEZO protein, much remains to be learned about how PIEZO interacts with other proteins to regulate its channel function and cellular signaling pathways.

Recent studies have identified several membrane proteins, namely TMEM120A (TACAN) (Beaulieu-Laroche et al. 2020), TMEM63 (OSCAs) (Murthy et al. 2018), TMEM87A (Elkin7) (Patkunarajah et al. 2020), and TMEM15°C (Tentonin3) (Hong et al. 2016), which were proposed to be mechanically activated at the cellular surface. However, their functional roles during mechanical activation and developmental physiology remain undefined. In the mouse model, null *TMEM120A* mutant mice exhibited reduced behavioral responses to mechanical stimuli (Beaulieu-Laroche et al. 2020). While TMEM120A was initially found to adapt mechanically activated (MA) currents in the Dorsal Root Ganglia (DRG) neuron (Beaulieu-Laroche et al. 2020), recent reports have debated this finding, suggesting that TMEM120A alone may not facilitate MA currents in different cell lines (Parpaite et al. 2021; Rong et al. 2021). Interestingly, when TMEM120A and PIEZO2 were co-expressed, there was a significant reduction in MA currents compared to the control cell background. This suggests that TMEM120A acts as a negative modulator of the PIEZO2 channel at the cell membrane (Del Rosario et al. 2022).

TMEM120A forms a homodimer complex, often accompanied by a coenzyme-A molecule (CoASH). This observation suggests that TMEM120A may affect fatty acid synthesis and lipid modification by regulating CoASH-dependent pathways (Niu et al. 2021; Rong et al. 2021; Xue et al. 2021). However, another structural study has raised questions about the presence of the CoASH in the human TMEM120A complex and has cast doubt on TMEM120A's functional role in lipid metabolism and modification (Chen et al. 2022). Overall, ongoing structural and electrophysiological studies have provided insights into the physiological functions of TMEM120. Nevertheless, further in vivo studies are needed to validate the proposed functions at the cellular and molecular levels. Using genetic model organisms will help uncover the genetic interactions between TMEM120A and potential determinants, such as PIEZOs.

To further understand the biological functions of TMEM120 (TACAN) in vivo, we functionally characterized *tmem-120* and *pezo-1*, the sole TACAN and PIEZOs orthologs in *Caenorhabditis elegans*. A fluorescent reporter allele *tmem-120::gfp* was expressed throughout development in *C. elegans*, with strong expression in the germline, embryos, and various somatic tissues. A full-length deletion allele, *tmem-120Δ*, led to multiple reproductive and fertility defects, including a reduced brood size, pinched zygotes, deformed germline, and maternal sterility. The reduced brood size was also observed in somatic or germline-specific *tmem-120* auxin-inducible degradation (AID) strains, underscoring the contributions of TMEM-120 from different tissues to reproduction. In vivo imaging revealed damaged or pinched zygotes in the uterus, indicating defects with passage through the spermatheca. Lastly, *pezo-1Δ*; *tmem-120Δ* double mutant restored the reduced brood size and alleviated defective sperm navigation behavior compared to the *pezo-1Δ* mutant only. In summary, these findings suggest that an epistatic interaction between *tmem-120* and *pezo-1* regulates reproduction in *C. elegans*.

## Materials and methods

*C. elegans* strains were maintained on MYOB (Modified Youngen's, Only Bacto-peptone) medium seeded with OP50*Escherichia coli* strain and grew in a 20°C incubator. The detailed strain information were indicated below: AG633 *tmem-120(av250) tmem-120::gfp CRISPR/Cas9 Edit*, knock-in GFP at C-terminus of *tmem-120*. AG634 *tmem-120 (av250) tmem-120::gfp; seip-1(av169) seip-1::mScarlet*. AG635 *tmem-120 (av250) tmem-120::gfp*; *ocfls2(pie-1p:mCherry::sp12::pie-1)*. AG632 *tmem-120(av251) tmem-120::::AID*. AG636 tmem-120::::AID; wrdSi23 [eft-3p::TIR1::F2A::mTagBFP2::AID*::NLS::tbb-2 3′UTR]. AG637 *tmem-120::*::AID; wrdSi50 [mex-5p::TIR1::F2A::mTagBFP2::AID*::NLS::tbb-2 3′UTR]. AG638 *tmem-120(av252) tmem-120Δ*, CRISPR/Cas9 Edit, knockout the full length of *tmem-120*. AG642 *tmem-120(av252) tmem-120Δ; pezo-1(av240) pezo-1Δ*. AG643 *tmem-120(av252) tmem-120Δ; pezo-1(av165) pezo-1(R2405P)*. AG570 *pezo-1(av240) pezo-1Δ* knockout the full length of *pezo-1*. AG742 *tmem-120 (av250) tmem-120::gfp; pezo-1(av142) mScarlet::pezo-1*.

### CRISPR design

The Bristol N2 strain was used as the wild type for CRISPR/Cas9 genome editing. A total of 20 nucleotide sequences of the *tmem-120* specific crRNAs were selected with the help of the customer crRNA design tool from Integrated DNA Technologies (IDT). All crRNA and tracRNA were synthesized by Horizon Discovery. The single-stranded donor oligonucleotides (ssODNs) of the *tmem-120* repair template were synthesized by IDT, and the detailed sequence information of the CRISPR reagents are listed in Supplementary Table 1. The 24-hour post-mid-L4 hermaphrodites (*n* = 20–30) were injected with mixed CRISPR/Cas9 reagents as described in the previous study (Iyer et al. 2019). All CRISPR/Cas9 gene-editing information are indicated in Supplementary Table 1. The putative-edited animals were then screened by PCR. The fluorescent signal of the *tmem-120::gfp* allele was imaged by a spinning disk confocal microscopy. The homozygous edited animals were then confirmed in subsequent generations. The GFP insertion animals were confirmed by confocal microscopy and Sanger sequencing.

### DAPI staining in *C. elegans*

Adult hermaphrodites were washed in a concavity slide filled with M9 buffer to remove the bacteria. Then, the cleaned hermaphrodites were transferred to 1 μl of egg white/M9/azide on SuperFrost slides (Daigger # EF15978Z); using eyelash to spread the egg white to a thin layer until all animals were stabilized on the slide. Slides were immediately immersed in a container of Carnoy's buffer to fix overnight (∼16 hours) at 4°C; rehydrating the fixed slides in different concentrations of ethanol (EtOH) for 2 minutes each, including 90% EtOH in water, 70% EtOH in water, 50% EtOH in PBS, 25% EtOH in PBS, and PBS alone. After the last wash, the slides were immersed in Coplin jars containing 1-μg/ml DAPI in PBS buffer for 10 minutes. DAPI-stained slides were then rinsed 3 times in PBS alone. Lastly, a drop of Vectashield mounting medium (#H-1500–10) was added to the slides; then, a coverslip was added on the top, followed by nail polish to seal the coverslip. Image acquisition was captured by a Nikon 60 × 1.2 NA water objective with 1-μm z-step size.

### Microscopy and imaging

All live imaging and DAPI staining slides were imaged by a spinning disk confocal microscope system, including an inverted Nikon Eclipse microscope, a Photometrics Prime 95B EMCCD camera, and a Yokogawa CSU-X1 confocal scanner unit. Images were acquired by Nikon's NIS imaging software using a Nikon 60 × 1.2 NA water objective. For DIC imaging, the tested animals were immobilized on 7% agar pads with an anesthetic (0.01% levamisole in M9 buffer). DIC image acquisition was performed using a Nikon 60 × 1.2 NA water objective with 1-μm z-step size; 15–20 planes were captured.

### Statistics

Statistical significance was determined by *P*-value from an unpaired 2-tailed *t*-test, and a 1-way ANOVA *t*-test. The Shapiro–Wilk and Kolmogorov–Smirnov Normality tests indicated that all data follow normal distributions.

## Results

### TMEM-120::GFP expressed in multiple tissues

**Fig. 1.**
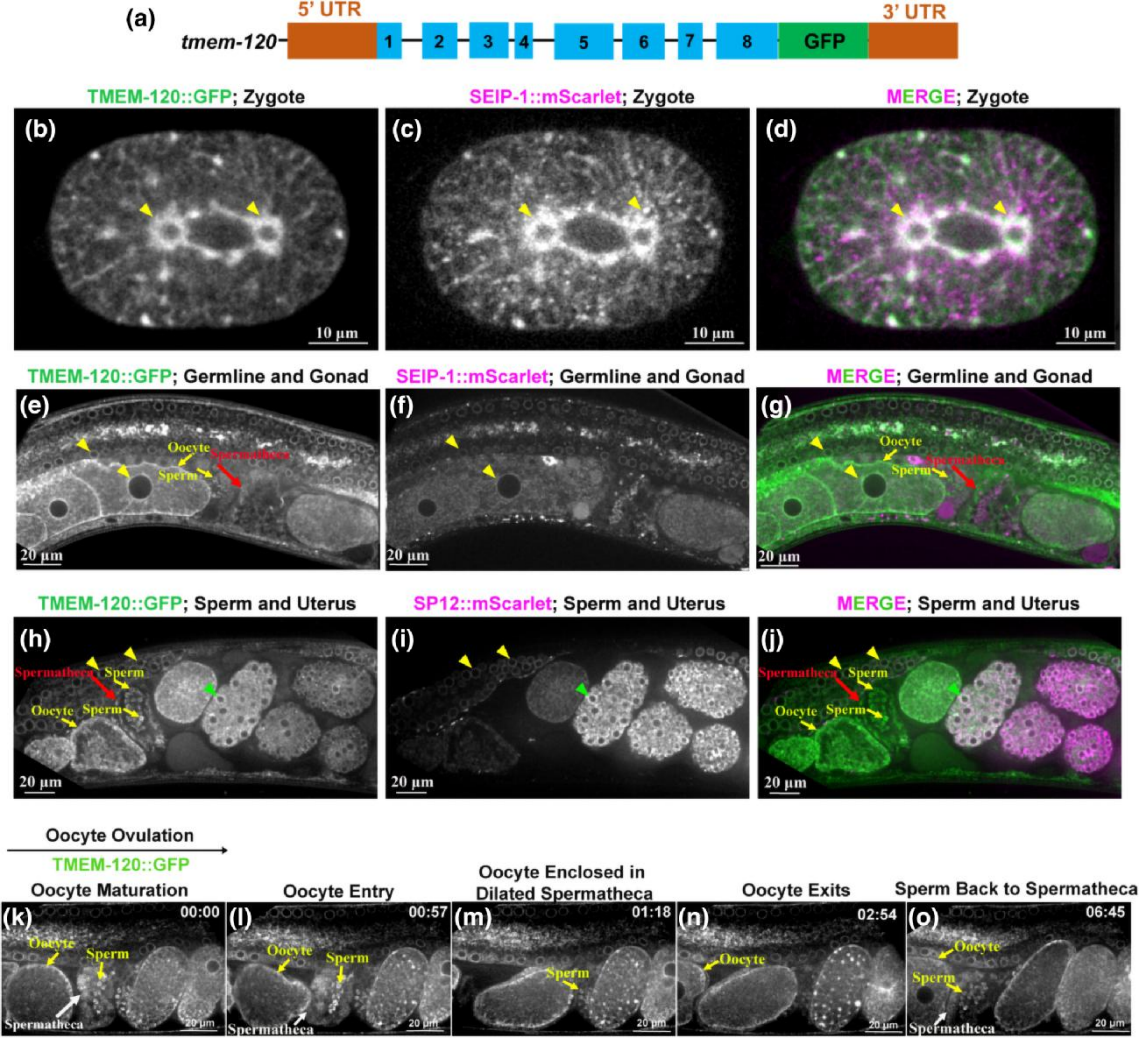
TMEM-120::GFP was expressed in multiple *C. elegans* tissues and cells. a) A green-fluorescent reporter gene *gfp* was knocked in at the C-terminus endogenous locus of *tmem-120* gene. b–d) TMEM-120::GFP (b, green in d) was strongly expressed at the ER network and perinuclei region (yellow arrowheads in b–d) in the 1-cell stage embryo. TMEM-120::GFP colocalized with a lipid droplet and ER dual marker SEIP-1::mScarlet (c, magenta in d). e–j) TMEM-120::GFP was also expressed at the perinuclei region in the germline (yellow arrowheads in e, g, h, and j), oocytes (yellow arrows in e and g), and multicell stage embryos (green arrowheads in h and j), which colocalized with ER markers SEIP-1::mScarlet (f, magenta in g) and SP12::mScarlet (i, magenta in j). k–o) Representative images of TMEM-120::GFP localization during ovulation and fertilization. TMEM-120::GFP (green) localized to the spermatheca (white arrow in k, l, and o), which remained closed before ovulation k and l). TMEM-120::GFP stably remained on the spermatheca before and after fertilization (k–o). TMEM-120::GFP was also present at the sperm membrane (yellow arrows in k–m, and o). The black arrow above the panel showed the orientation when the embryos traveled through the spermatheca from left to right. The time of each step is labeled on the top right in minutes and seconds. Scale bars are indicated in each panel.

The *C. elegans* genome encodes a single *TMEM120A/B* ortholog, *tmem-120*. The *C. elegans* TMEM-120 protein shares ∼42 and 41% identity to human TMEM120A and TMEM120B, respectively (Supplementary Fig. 1a). To visualize the expression pattern of *tmem-120* in vivo, we knocked in a green-fluorescent reporter gene *gfp* at the endogenous C-terminus of *tmem-120* using CRISPR/Cas9 editing (Fig. 1a). The genome-edited *tmem-120::gfp* are superficially wild type, suggesting that the GFP knock-in did not disrupt TMEM-120 function in vivo. TMEM-120::GFP is globally expressed in *C. elegans*, including embryos (Fig. 1, b, d, h, and j), germline cells (Fig. 1, e, g, h, and j), multiple somatic tissues including expression at spermatheca (red arrows in Fig. 1, e, g, h, and j, white arrows in Fig. 1, k, l, and o) and muscle (yellow arrowheads in Supplementary Fig. 1b). Under higher magnification, TMEM-120::GFP was observed to co-localize with a lipid droplet and endoplasmic reticulum (ER) marker SEIP-1::mScarlet (Fig. 1, c, d, f, and g) and another ER maker SP12::mCherry (Fig. 1, i and j) at the perinuclear region (yellow arrowheads in b–j, green arrowhead in the late embryos, h–j), suggesting that TMEM-120::GFP is an ER network resident protein. The expression pattern of TMEM-120::GFP at ER structure was consistent with a preprint report (LI et al. 2021) in *C. elegans* and another study of *TMEM120A* in mammalian cells (Batrakou et al. 2015). Live imaging and detailed analysis of TMEM-120::GFP expression pattern during reproduction also revealed that TMEM-120::GFP was expressed on the oocyte and sperm membrane (yellow arrows in Fig. 1, e, g, h, j, and k–o, and Supplementary Video 1). We also observed the expression of TMEM-120::GFP in spermathecal cells (red arrows in e, g, h, and j, white arrow in k–l and o), suggesting that TMEM-120 may regulate spermathecal contraction and dilation during ovulation (Fig. 1k–o, Supplementary Video 1). Additionally, TMEM-120::GFP-labeled sperm migrate back to the constricting spermatheca after each ovulation completes after the fertilized oocyte is compelled into the uterus (Fig. 1o, and Supplementary Video 1). Collectively, our data indicated that TMEM-120::GFP is an ER resident protein and was expressed in different somatic cells, germline cells, and sperm in *C. elegans*.

### Deletion of TMEM-120 caused maternal sterility and reduced brood size

**Fig. 2.**
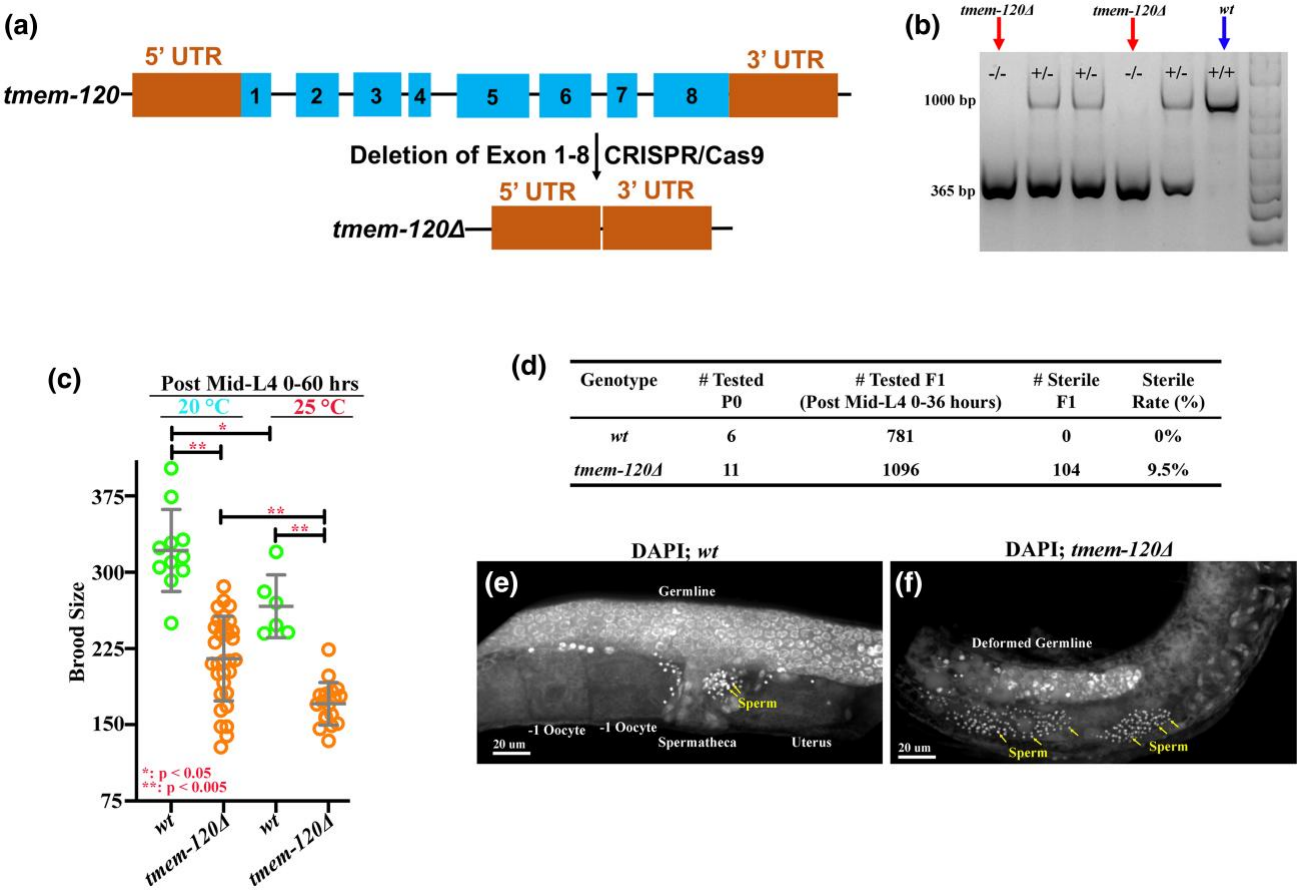
Deletion of *tmem-120* gene caused sterile animals and reduced brood size. a) Schematic of the *tmem-120Δ* full-length deletion allele, *tmem-120Δ*, by CRISPR/Cas9. b) Representative PCR gel from genotyping single animals for *tmem-120Δ* knockout candidate lines. A positive homozygous knockout line is labeled with (−/−) (red arrows in b). c) Brood size was significantly reduced in *tmem-120Δ* animals compared to wild type, and this reduction was enhanced at a higher temperature (25°C). d) Approximately 10% of the sterile animals were found in the *tmem-120Δ* F1 population when compared with wildtype control. e and f) DAPI staining demonstrated that the sterile *tmem-120Δ* animals bear deformed and shorter germline f) than the wildtype control e), while sperm were present in *tmem-120Δ* mutant uteri (yellow arrows in f). Scale bars are indicated in each panel. *P < 0.05; **P < 0.005 c).

To further characterize the functional roles of *tmem-120* in *C. elegans*, a full-length deletion allele, *tmem-120Δ*, was generated by CRISPR/Cas9 editing, and the mutant phenotypes were examined (Fig. 2, a and b). The entire *tmem-120* coding sequence, including 8 exons and 7 introns, was deleted in the null animals (Fig. 2, a and b). Although TMEM-120::GFP was expressed widely in the germline cells and embryos, the embryonic viability in the homozygous *tmem-120Δ* mutants is near 100%, which is identical to wildtype control at both 20°C and 25°C (Supplementary Fig. 2a). However, the number of F1 progeny was significantly reduced compared to wild type (Fig. 2c). The reduction of brood size was enhanced when animals were grown at a higher temperature (25°C) (Fig. 2c). In addition, about 10% of F1 larvae are sterile in the *tmem-120Δ* homozygous population (Fig. 2d). DAPI staining showed a deformed germline observed in these sterile *tmem-120Δ* animals, and sperm (yellow arrows in Fig. 2f) were spread throughout the germline (Fig. 2f) instead of residing in the spermatheca as shown in the wildtype animals (yellow arrows in Fig. 2e). These observations suggested that proper spermatogenesis was not disturbed in the *tmem-120Δ* mutants. However, the loss of *tmem-120* is sufficient to affect germline morphogenesis and oogenesis, which led to deficient reproduction in *C. elegans*. Given that TMEM-120::GFP was expressed on the sperm membrane (Fig. 1, h, j, k–m, and o, and Supplementary Video 1), it is plausible that small brood size in the *tmem-120Δ* mutant is due to impaired sperm mobility or sperm fertility. To test for the ability of sperm to fertilize oocytes, both *tmem-120Δ* mutant and wildtype males were mated with female *fem-1(hc17)* animals, which do not produce self-sperm (Supplementary Fig. 2b). The unmated *fem-1(hc17)* animals did not have any viable progeny (Supplementary Fig. 2b and c), and only unfertilized oocytes were laid on the plate at the nonpermissive temperature (25°C) (Supplementary Fig. 2b and c). The mated *fem-1(hc17)* animals produced cross-progeny after mating with both *tmem-120Δ* mutant and wildtype males (Supplementary Fig. 2b and c), indicating that *tmem-120Δ* mutant sperm are functional and that their sperm is capable of migrating through the uterus to the spermatheca for fertilization.

#### Tissue-specific degradation of TMEM-120 reveals multiple roles of TMEM-120 in both somatic tissues and germline to *C. elegans* reproduction

**Fig. 3.**
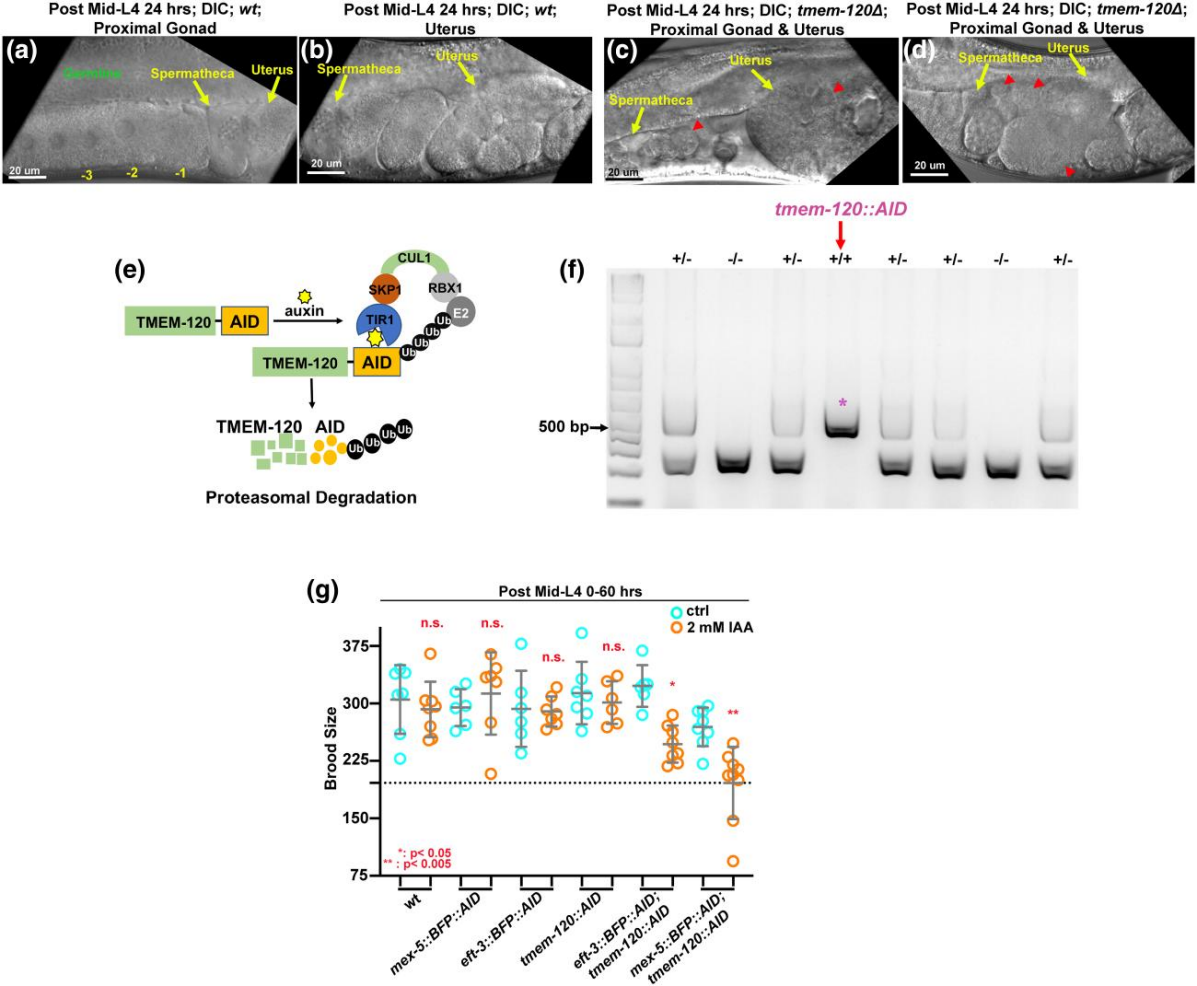
*tmem-120Δ* mutant showed pinched and damaged zygotes in the uterus. a and b) Representative images of the aligned oocytes in the wildtype proximal gonad a) and the zygotic embryos in the wildtype uterus after fertilization b). c and d) In contrast, the pinched zygotes were left in the *tmem-120Δ* mutant spermatheca and uterus (red arrowheads in c and d). e) A degron tag was knocked in at the *tmem-120* C-terminus endogenous locus using CRISPR/Cas9. f) Representative PCR gel from genotyping single animals for *tmem-120::AID* knock-in candidates. A positive homozygous knock-in line is labeled with a red asterisk. g) Brood sizes were reduced in each degron strain when animals were exposed to 2-mM auxin IAA. *P < 0.05; **P < 0.005.

We also observed that pinched zygotes were frequently observed in the *tmem-120Δ* uterus and spermatheca (red arrowheads in Fig. 3, c and d), while intact zygotes were observed in wildtype proximal uterus (Fig. 3, a and b), suggesting that the post-ovulation zygotes were damaged during spermathecal transit. Multiple reproductive tissues contribute to proper ovulation in *C. elegans*, including the somatic sheath, spermatheca, and germline cells. Sheath cells and spermathecal cells contract to push the oocyte through the spermatheca, while germline cells secret chemical signals such as prostaglandins to initiate the sheath and spermatheca contraction or dilation and guide the self-sperm in the uterus to migrate back to the spermatheca after each ovulation. To dissect the functional contributions of TMEM-120 in different reproductive tissues, we utilized an AID system to degrade TMEM-120 in all somatic tissues or in germ line cells (Fig. 3e). We first knocked in the degron coding sequence at the *tmem-120* C-terminus endogenous locus by CRISPR/Cas9 editing. The allele was named *tmem-120::AID* (Fig. 3, e and f). To activate the AID system, the *tmem-120::AID* strain was crossed with the strains expressing the degron interactor transgene *tir-1::BFP::AID* driven by P*eft-3* or P*mex-5* promoter. *Peft-3::tir-1::BFP::AID* was expressed in most or all somatic tissues, while *Pmex-5::tir-1::BFP::AID* was expressed in the germline. *tir-1::BFP::AID* strains were also tagged with a cassette with both blue fluorescent reporter and degron coding sequence, which was used as a marker for auxin exposure. To characterize the defects associated with the degradation of TMEM-120 in these different tissues, L4 animals were exposed and maintained on 2-mM auxin medium or 0.25% ethanol as a control for 1 generation, and the brood size was determined 0–60 hours post-mid L4. Interestingly, the brood size was significantly reduced in each *tmem-120::AID* strain when compared with the control, regardless of the promoters used (Fig. 3g), while the reduction in brood size was more severe in the germline *Pmex-5::tir-1::BFP::AID* strain. These data suggest that multiple inputs of TMEM-120 from different tissues regulate reproduction.

### The sub-sterility of *pezo-1Δ* mutant was alleviated upon deletion of *tmem-120*

**Fig. 4.**
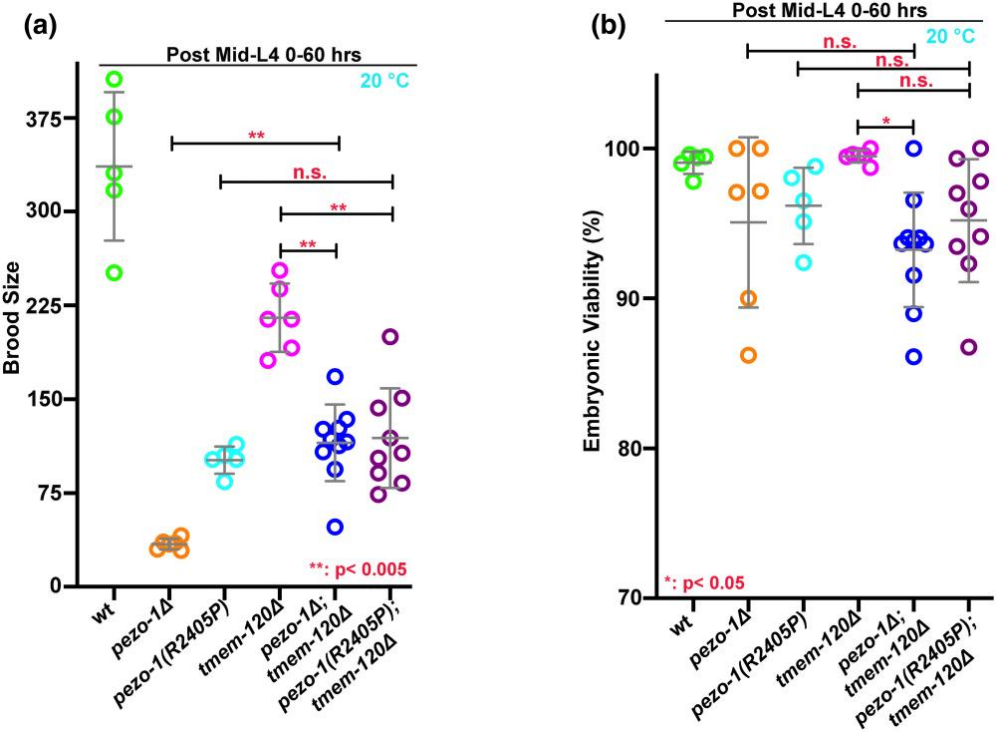
*tmem-120Δ* show genetic interactions with *pezo-1*. a and b) *tmem-10Δ* alleviated the reduced brood size in *pezo-1Δ* animals but not in *pezo-1(R2405P)*, a putative gain-of-function mutant. b) The embryonic lethality was not significantly affected in *tmem-10Δ*; *pezo-1Δ* double mutants when compared with *pezo-1Δ* only. *P < 0.05 b); **P < 0.005.

TMEM-120 is the ortholog of the mammalian mechanosensitive channel TACAN/TMEM120A (Beaulieu-Laroche et al. 2020; Davis et al. 2022), although its role in mechanotransduction is debated. A recent report suggested that TMEM-120 may be a negative modulator to regulate PIEZO2 when triggering MA current at the cellular surface (Del Rosario et al. 2022). In this study, we tested whether there was a genetic interaction between *tmem-120Δ* mutant and *pezo-1* mutants, including *pezo-1Δ*, and a putative gain-of-function missense allele *pezo-1(R2405P)* in vivo. The dual null alleles of *tmem-120Δ* and *pezo-1Δ* (*n* = 115.2) alleviated the small brood size in *pezo-1Δ* alone (*n* = 34.2) (Fig. 4a), while *tmem-120Δ* did not show repress the reduced brood size in the *pezo-1(R2405P*) mutant. Additionally, neither enhanced nor repressed embryonic lethality was observed in the *pezo-1Δ* and *tmem-120Δ* double mutant compared with *pezo-1Δ* only (Fig. 4b). Therefore, our genetic study indicated that an epistatic interaction between *tmem-120* and *pezo-1* during *C. elegans* reproduction.

We then visualized the colocalization pattern of *tmem-120* and *pezo-1* by imaging a strain expressing both *tmem-120::gfp* and *mScarlet::pezo-1* reporter alleles (Supplementary Fig. 3a–o). TMEM-120::GFP and mScarlet::PEZO-1 co-expressed at the *C. elegans* pharyngeal–intestinal valve (red arrows in Supplementary Fig. 3a–c), which underwent consistent mechanical stimuli during food intake. TMEM-120::GFP also expressed at germline perinuclei (green arrows in Supplementary Fig. 3g and i) and the oocyte membrane (red rectangles in Supplementary Fig. 3g and i; yellow arrows in j and k), which colocalized with mScarlet::PEZO-1 (yellow arrows in Supplementary Fig. 3j and l). In *C. elegans*, the sperm resides in a somatic myoepithelial tissue, spermatheca, in which the plasma membrane was labeled by mScarlet::PEZO-1 (yellow rectangles in Supplementary Fig. 3h and i, green arrows in Supplementary Fig. 3m and n), while TMEM-120::GFP is primarily expressed in the spermathecal cytosol (yellow rectangles in Supplementary Fig. 3g and i, red arrows in Supplementary Fig. 3m and o). Collectively, our data indicated that TMEM-120::GFP and mScarlet::PEZO-1 co-expressed in multiple mechanical stimuli tissues, including pharyngeal–intestinal valve and spermathecal cells, suggesting that both proteins may coordinate to regulate the *C. elegans* reproduction and other mechanical stimuli processes.

### Sperm navigation back to spermatheca was disrupted in *pezo-1Δ* mutant but was significantly alleviated by *tmem-120Δ*

In wildtype hermaphrodites, sperm are expelled from the uterus along with the fertilized zygote. These dispersed sperm then swiftly return to the spermatheca through the spermathecal-uterine valve. Proper sperm migration is guided by F-series prostaglandins derived from polyunsaturated fatty acid (PUFAs), which is secreted from oocytes and somatic sheath cells (Miller 2001; Miller et al. 2003; Kubagawa et al. 2006; Han, Cottee and Miller 2010). Our prior studies demonstrated that *pezo-1* mutants caused defective sperm migration back to the spermatheca, causing sperm retention in the uterus (Bai et al. 2020, 2023).

**Fig. 5.**
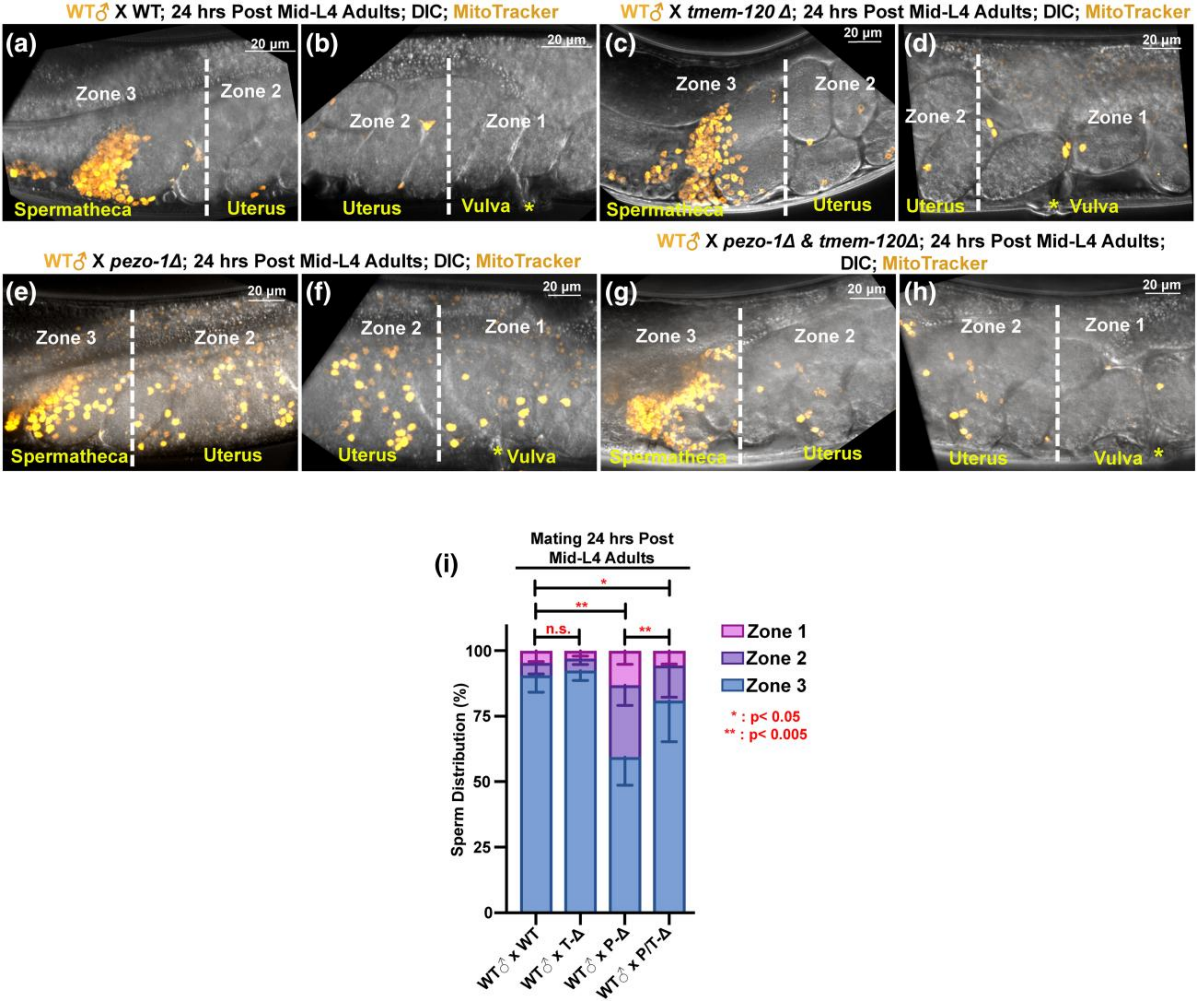
Sperm guidance and navigation were disrupted in *pezo-1Δ* mutant, but were significantly rescued by *tmem-120Δ*. a) The sperm migration was quantified by counting the sperm in 3 different zones, including Zone 1 (the area closest to the vulva), Zone 2 (uterus), and Zone 3 (the area is adjacent to the spermatheca and +1 fertilized embryos). a–h) The distribution of fluorescent male sperm was labeled with MitoTracker in each zone in wildtype, *tmem-10Δ*, *pezo-1Δ*, and *tmem-10Δ*; *pezo-1Δ* double mutants. Yellow asterisks indicate the vulva b, d, f, and h). Scale bars were indicated in each panel. i) Quantification of sperm distribution percentage values in each zone. *P < 0.05; **P < 0.005.

To assess whether *tmem-120* contributes to sperm attraction in *pezo-1Δ* mutant, we mated male animals with each mutant hermaphrodite to evaluate sperm navigational performance and distribution in vivo. The mating males were labeled with a vital fluorescent dye, MitoTracker CMXRos, which efficiently labeled the sperm mitochondria in live animals (Whitten and Miller 2007). All tested wildtype males were mated with unstained hermaphrodites for a minimum of 30 minutes. The uterus was divided into three zones to assess and quantify the sperm distribution (McKnight et al. 2014). Sperm counts were conducted in each zone one hour after the stained males were removed from the mating plates. In wildtype hermaphrodites, over 90% of fluorescent sperm successfully navigated through the uterus and accumulated in Zone 3 (Fig. 5, a and i), the zone closest to the spermatheca. Only a few fluorescent sperm were observed in Zone 1 and Zone 2 (Fig. 5, b and i), the zones nearest to the vulva, indicated by an asterisk mark (Fig. 5, b, d, f, and h). However, in Day One *pezo-1Δ* adult hermaphrodites, approximately 45% of the fluorescent male sperm failed to reach the spermathecal Zone 3 (Fig. 5, e, and i), with the remaining sperm distributed throughout Zone 1 and Zone 2 (Fig. 5, e, f, and i). These findings were consistent with previous studies on other *pezo-1* null and gain-of-function mutants (Bai et al. 2020, 2023).

Interestingly, the sperm navigation defects were mitigated in the *pezo-1Δ*; *tmem-120Δ* double mutant (Fig. 5g–i), with approximately 75% of the stained sperm successfully returning to Zone 3 and less than 25% distributed in Zone 1 and Zone 2 (Fig. 5g–i). In contrast, we did not observe significant sperm navigation deficiencies back to spermatheca in the *tmem-120Δ* single mutant (Fig. 5, c, d, and i). In summary, all tested male sperm exhibited normal motility and navigation in wildtype and *tmem-120Δ* hermaphrodites. However, in *pezo-1Δ* hermaphrodite reproductive tracts, wildtype sperm displayed defects in sperm navigational behavior and a reduced ability to crawl toward the spermatheca. This defective sperm navigational behavior was significantly improved in the *pezo-1Δ*; *tmem-120Δ* double mutant, suggesting that *tmem-120* positively interacts with *pezo-1* to coordinate the proper sperm attraction signal during reproduction.

## Discussion

TMEM120A (TACAN) was proposed to play a crucial role in activating current mechanically and regulating lipid modification and metabolism through a CoASH-dependent pathway (Beaulieu-Laroche et al. 2020; Rong et al. 2021; Xue et al. 2021), yet the exact molecular mechanisms remain debated and largely uncharacterized. This study presents in vivo and genetic analysis of the only ortholog of *TMEM120A*/*TACAN*, *tmem-120*, in *C. elegans*. We focused on the reproductive system because of significant brood reduction and germline defects in the *tmem-120* mutant. The *C. elegans* reproductive cycle contains multiple steps, including oocyte maturation, ovulation (matured oocytes entry and exit from the spermatheca), fertilization, and sperm navigation back to the spermatheca after each ovulation cycle. Utilizing the tissue-specific degradation of TMEM-120, our data demonstrated that dysfunction of *tmem-120* in either somatic tissue or germline caused a significantly reduced brood size, suggesting that TMEM-120 from different tissues contributed to proper reproduction. Ovulation is a mechanically activated process incorporated by multiple somatic tissues, including gonadal sheath cells, spermathecal body cells, and distal spermathecal valve. After fertilization, the fertilized zygote was expulsed into the uterus driven by spermathecal contraction and the gating of the spermatheca-uterine valve. We observed pinched zygotes in the uterus of the *tmem-120* mutant, suggesting that the loss of *tmem-120* caused dramatic effects on the ovulation and zygote expulsion from the spermatheca.

This study also revealed that loss of *tmem-120* significantly alleviated sperm attraction deficiency in the *pezo-1* mutant. *C. elegans* oocytes and somatic sheath cells synthesized and secreted lipophilic hormones F-series prostaglandins into the reproductive tract to attract sperm navigating back to the spermatheca after each fertilization event (Miller 2001; Edmonds et al. 2010; Han, Cottee and Miller 2010; Hoang et al. 2013). Our previous report showed that the severe deficiency of sperm navigation back to the spermatheca in the *pezo-1* mutant was likely due to lipophilic hormone signaling deficient instead of sperm migration deficiency (Bai et al. 2020). The structural studies of TMEM120A indicated that TMEM120A forms a helical barrel where a CoASH is notable for its functional roles in fatty acid synthesis and modification (Niu et al. 2021; Rong et al. 2021; Xue et al. 2021). These insightful studies implied that TMEM120A may be involved in fat metabolism by its hypothetical enzymatic function. TMEM120A was also suggested to negatively regulate PIEZO2 to reduce the mechanically activated current by modifying the lipid content of the cell (Del Rosario et al. 2022). Moreover, a recent study demonstrated that adipocyte-specific *TMEM120A* knockout mice displayed severe disruption in genome organization and fat metabolism in vivo, which led to a distinct latent lipodystrophy pathology (Czapiewski et al. 2022). Lastly, a recent preprint study suggested that *tmem-120* may regulate the fatty acid intake in *C. elegans*, and loss of *tmem-120* led to a reduction of triacylglycerol level (LI et al. 2021). Therefore, we hypothesized that *C. elegans tmem-120* might have a conserved function in regulating fatty homeostasis and lipid metabolism. The loss of *tmem-120* may affect the lipid metabolism or fatty acid synthesis, precursor of prostaglandins in *C. elegans*, to compromise the defective sperm navigation behaviors in *pezo-1* mutants. The alleviation of sperm navigation defects in *tmem-120* and *pezo-1* double mutant could also directly contribute to restoring the small brood size in the double mutant when compared to the *pezo-1* mutant only.

In summary, we utilized the facile *C. elegans* reproductive tract with tubular tissue and valves mechanically activated by oocytes during ovulation and fertilization to study *tmem-120* and *pezo-1* in vivo. We have demonstrated that the *C. elegans tmem-120* is required for efficient reproduction, germline formation, and its genetic interaction with another mechanosensory molecule, *pezo-1* mutant, with the brood size as a readout. The reduction in brood size that we observed in the *tmem-120* mutant will allow us to understand further the contribution of *tmem-120* during mechanotransduction and other physiological processes. The effect in sperm navigation performance we observed in *tmem-120*; *pezo-1* double mutant, further implied that *tmem-120* might regulate the prostaglandins precursor polyunsaturated fatty acid synthesis or modification in *C. elegans*. Future studies will determine whether and how *tmem-120* contributes to lipid modification and synthesis, possibly contributing to prostaglandin synthesis and sperm navigation deficiency. Lastly, the *C. elegans* reproductive tract and sperm attraction model may provide a new tool to investigate the potential functions of TMEM120A, such as utilizing the CoASH as a substrate or interactor to regulate fat metabolism, which await to be explored in the future.

## Supplementary Material

jkad251_Supplementary_Data

## Data Availability

The *C. elegans* strains are available upon request. Supplementary Figs. 1–3 and Supplementary Video 1 are available on G3. The authors affirm that all data necessary to confirm the article's conclusions are present in the article, figures, videos, and tables. The genotype and phenotype data of *tmem-120* and *pezo-1* mutants will be submitted to WormBase.org. Supplemental material available at G3 online.
