# Supplementary material for: Transmembrane protein 120A (TMEM-120A/TACAN) coordinates with PIEZO channel during Caenorhabditis elegans reproductive regulation
Source: G3 (Bethesda). 2023 Dec 5;14(1):jkad251. doi: 10.1093/g3journal/jkad251 (PMC10755168; doi:10.1093/g3journal/jkad251)
Supplement: jkad251_Supplementary_Data [file jkad251_supplementary_data.zip › Supplemental_Figure_and_Video_Legend_G3-2023-404545.docx]

**Figure S1 *C. elegans* TMEM-120 was conserved with human TMEM120A and TMEM120B.**

(A) Sequence alignment of *C. elegans* TMEM-120 with human TMEM120A and TMEM120B. (B) TMEM-120::GFP was expressed in the muscular tissue (yellow arrowheads in B). The scale bar was indicated on the bottom left.

**Figure S2 Sperm is fertile in the *tmem-120Δ* mutant.**

(A) The percentage of viable embryos was not affected in the *tmem-120Δ* animals. (B-C) Both *tmem-120∆* and wildtype males are fertile and sire progeny when mated with *fem-1(hc17ts)* mutants (essentially female animals). (C) No significant embryonic lethality was observed in the F1 progenies after mating.

**Figure S3 Co-expression of TMEM-120::GFP and mScarlet::PEZO-1 in *C. elegans*.**

(A-F) TMEM-120::GFP (A, green in C) was strongly expressed at the pharyngeal-intestinal valve (red arrows, A-C), which overlapped with mScarlet::PEZO-1 (B, magenta in C). (D-F) The enlarged pictures of the yellow rectangle area indicated the colocalization of mScarlet::PEZO-1 (E, magenta in D) and TMEM-120::GFP (F, green in D). (G-I) Colocalization of mScarlet::PEZO-1 (H, magenta in I) and TMEM-120::GFP (G, green in I) on the oocyte plasma membrane (red box in G-I) and spermathecal cells (yellow box in G-I­). Green arrows indicated that mScarlet::PEZO-1 labeled gonadal membrane and TMEM-120::GFP expressed at the germline nuclei membrane. Red arrows indicated that mScarlet::PEZO-1 and TMEM-120::GFP were expressed in the spermathecal cells. (J-L) The enlarged picture of the red box in G-I showed colocalization of mScarlet::PEZO (L, magenta in J) and TMEM-120::GFP (K, green in J) on the oocyte plasma membrane (yellow arrows). (M-O) The enlarged images of the yellow box in G-I. TMEM-120::GFP and mScarlet::PEZO-1 were observed in the spermathecal tissue, TMEM-120::GFP was expressed in the spermathecal cytosol (red arrows in M and O), while mScarlet::PEZO-1 was expressed at the spermathecal membrane (green arrows in M-N). Yellow arrows in M-N indicate that the sperm reside inside the spermatheca. The scale bars were labeled in each panel.

**Video S1 TMEM-120::GFP expression pattern during ovulation.**

Ovulation imaged in the genome-edited animals expressing TMEM-120::GFP. Images are single z-plane taken every 3 s. The playback rate is 10 frames/second.
