# Supplementary material for: Transmembrane protein 120A (TMEM-120A/TACAN) coordinates with PIEZO channel during Caenorhabditis elegans reproductive regulation
Source: G3 (Bethesda). 2023 Dec 5;14(1):jkad251. doi: 10.1093/g3journal/jkad251 (PMC10755168; doi:10.1093/g3journal/jkad251)
Supplement: jkad251_Supplementary_Data [file jkad251_supplementary_data.zip › Table_S1_G3-2023-404545.docx]

**Table S1 List of the sequence for the CRISPR design**

| **Strain** | **Genotype** | **Description** | **Sequence Name** | **Sequence 5’-3’** | **PAM** |
| --- | --- | --- | --- | --- | --- |
| AG638 | *tmem-120 (av253)* III*.* | Deletion of exons 1-8 and 1-7 introns of *tmem-120* | crRNA N-terminus | gatttttttgcagATGGCGA | CGG |
|  |  |  | crRNA C-terminus | TCAGTGCAAATGAAGTTTTC | CGG |
|  |  |  | Repair Template | aaatccgaatttttttatcgatttttttgcagATGTGAgacgacgatttgcataattttatgtttttttg | |
|  |  |  | Genotyping F1 | gctagtacaccacactgcgt | |
|  |  |  | Genotyping R1 | ttctggttcccagcttaccg | |
|  |  |  | Genotyping  Internal | tggttcgggcctttcagata | |
| AG633 | *tmem-120 (av250 [tmem-120::gfp])* III*.* | Knock in GFP at C-terminus of *tmem-120,* GFP was amplified from plasmid pDD282 | crRNA | TCAGTGCAAATGAAGTTTTC | CGG |
|  |  |  | Repair Template F1 | G AGCCGCTGCTACGTGGAGCTCCGCCACCGCCA AC**A** GG**T** AA**G** CT**C** CA**C** TT**A** CA**T** GGAGCATCGGGAGCC (GFP) | |
|  |  |  | Repair Template R1 | caaaaaaacataaaattatgcaaatcgtcgtcTCACTTGTAGAGCTCGTCCATTC (GFP) | |
| AG632 | *tmem-120 (av251 [tmem-::degron])* III | Knock in Degron sequence at C-terminus of *tmem-120.* Degron was amplified from plasmid pK0132 | crRNA | TCAGTGCAAATGAAGTTTTC | CGG |
|  |  |  | Repair Template F1 | GAGCCGCTGCTACGTGGAGCTCCGCCACCGCCA AC**A** GG**T** AA**G** CT**C** CA**C** TT**A** CA**T** ggagcatcgggagcctcaggagcatcg (linker)GACTACAAAGACCATGACGGTG (Degron) | |
|  |  |  | Repair Template R1 | caaaaaaacataaaattatgcaaatcgtcgtcTCA CTTCACGAACGCCGCC (Degron) | |

**Note: Capital letters represent the ORF or exon sequence, small letters indicate the sequence from intron. Bolded letters indicate the optimized base needed for the CRISPR design.**
