# Supplementary figures and images for: Transmembrane protein 120A (TMEM-120A/TACAN) coordinates with PIEZO channel during Caenorhabditis elegans reproductive regulation
Source: G3 (Bethesda). 2023 Dec 5;14(1):jkad251. doi: 10.1093/g3journal/jkad251 (PMC10755168; doi:10.1093/g3journal/jkad251)

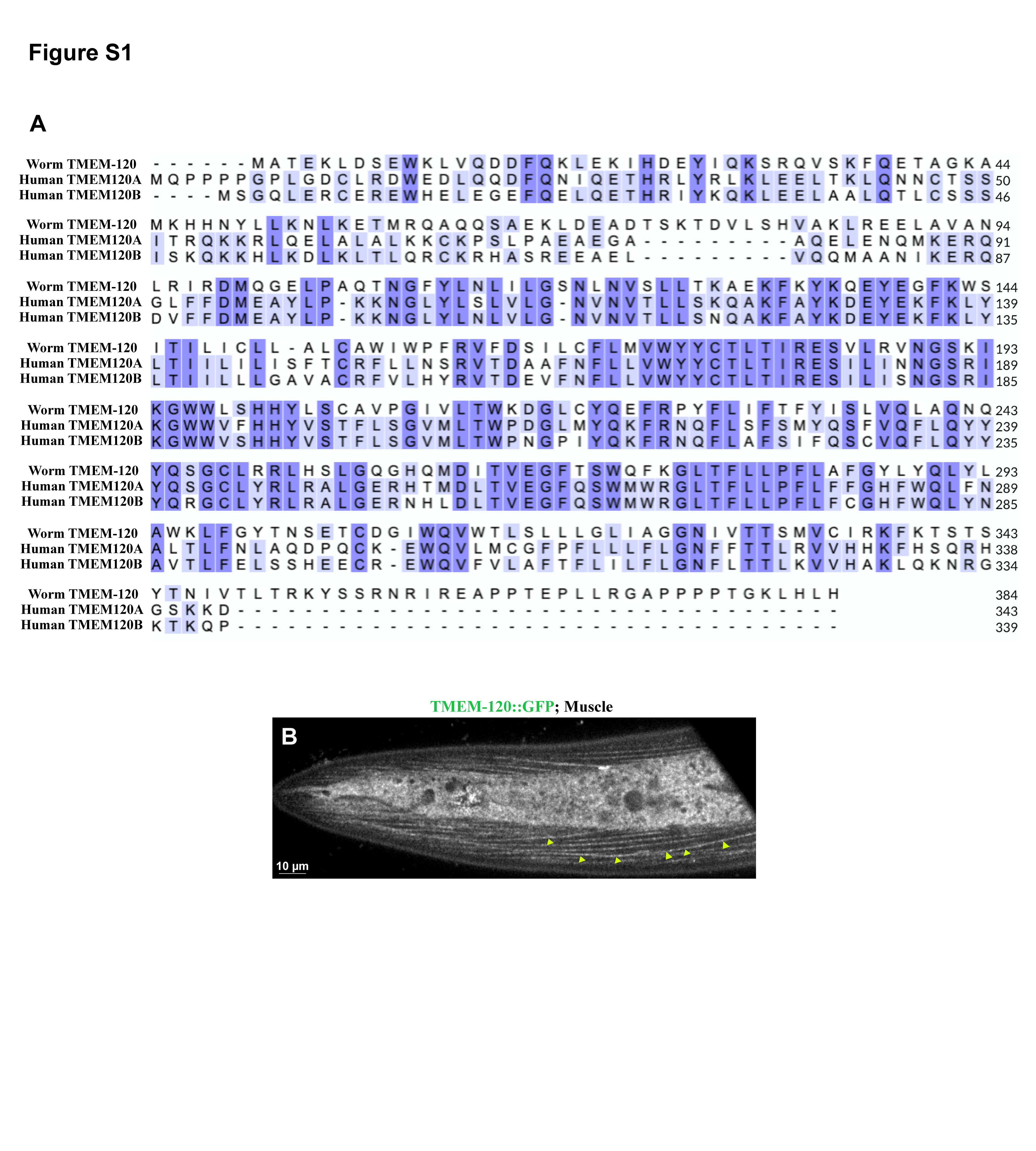

Supplement: jkad251_Supplementary_Data [file jkad251_supplementary_data.zip › Figure_S1_G3-2023-404545.tif]

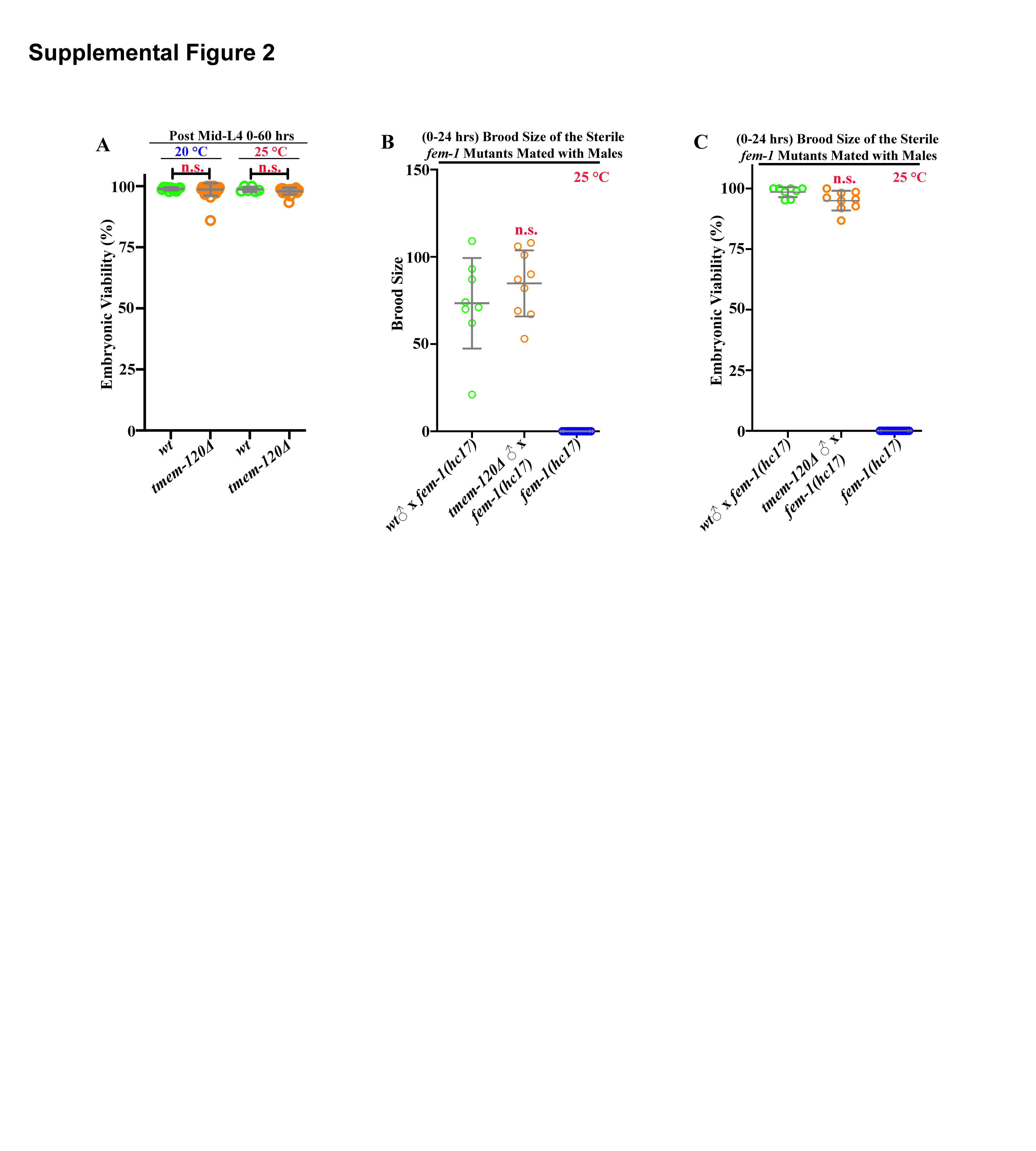

Supplement: jkad251_Supplementary_Data [file jkad251_supplementary_data.zip › Figure_S2_G3-2023-404545.tif]

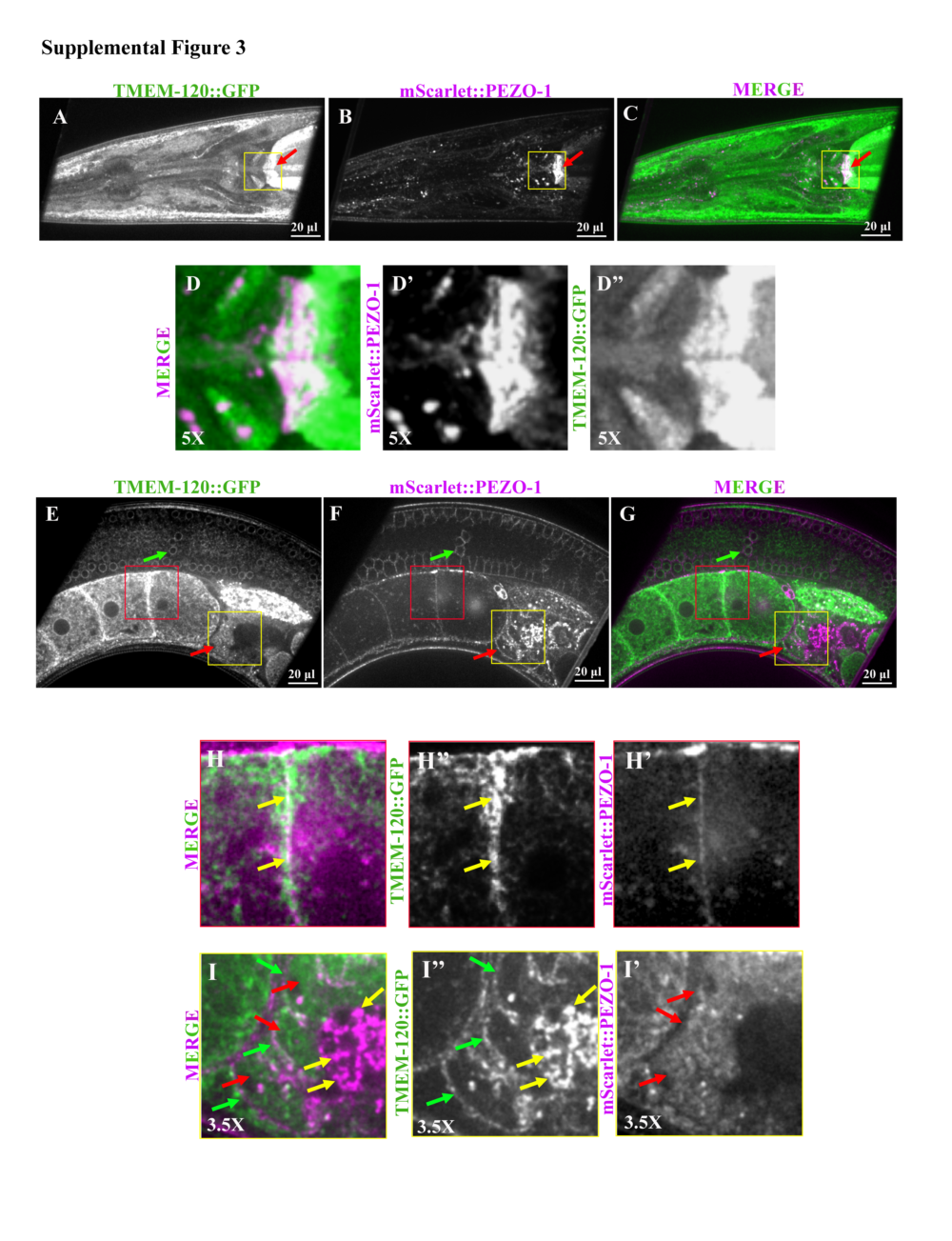

Supplement: jkad251_Supplementary_Data [file jkad251_supplementary_data.zip › Figure_S3_G3-2023-404545.tif]
